# Skin-to-skin contact: multicultural perspectives on birth fluids and birth ‘dirt’

**DOI:** 10.1111/inr.12100

**Published:** 2014-04-09

**Authors:** V Finigan, T Long

**Affiliations:** 1 Consultant Midwife-Infant Feeding, Antenatal Clinic, Pennine Acute NHS Hospitals Trust, Royal Oldham HospitalOldham; 2 Professor of Child and Family Health, School of Nursing, Midwifery and Social Work, University of SalfordSalford, UK

**Keywords:** Breastfeeding Care, Maternity, Family Health, Multicultural Issues, Culture, Parenting, Family Health, Phenomenology, Research, Post-Partum Care

## Abstract

**Aim:**

To explore the experiences of women from three population groups of immediate skin-to-skin contact (SSC) with their newborn babies.

**Method:**

A mixed methods approach was adopted in a phenomenological study to elicit the experiences of English, Pakistani and Bangladeshi women. Audiotaped diaries, semi-structured interviews, photographs and video recordings were employed. Concept mapping was central to data analysis.

**Results:**

This paper reports novel findings that women contextualized and accepted secretions and bodily fluids from birth. This contradicts the beliefs of midwives that Asian women find bodily secretions abhorrent and culturally unacceptable. All participants reported positive experiences of SSC despite varying degrees of soiling from birth fluids.

**Limitations:**

The study was conducted in a single setting, and participants may not have been representative of others in their cultural groups. Third-party translation may have added an unsought layer of interpretation. The imposition of cultural expectations by peers in the recruitment process excluded some potential participants.

**Conclusion:**

Stereotypical assumptions about cultural background often characterize professional responses. When this stereotyping was put aside, women of all three cultures, whether breastfeeding or bottle-feeding, were able to enjoy SSC with their babies.

**Implications for Nursing and Health Policy:**

The findings suggest that changes will be needed in professional practice to be more open to women's expressed preferences, in local policy to ensure that choices are made clear and are available, and in national strategic direction to ensure widespread adoption of positive practices for opportunities to increase breastfeeding, promote parent–child bonding and support patient choice to be realized.

## Introduction

In the UK, policies have been introduced to reform maternity services, aiming to plan and deliver care that is more responsive to the needs of individual women and their families (Royal College of Midwives (RCM) 2008). The intention was to offer informed choice on all aspects of care including the right to choose skin-to-skin contact (SSC) at birth. The RCM recognized that where women-centred care underpins midwives' practices, women will become empowered, drawing on their own strengths, power and skills.

Changing embedded beliefs and ritualized care is challenging and even more difficult to address when evidence to support the change is limited. This study was driven by a desire to fill a gap in knowledge about women's experience of SSC from diverse population groups. Ultimately, the goal was to answer a practical problem and to justify a change in midwifery practice. The women's own voices recounting their experiences were used to shape midwifery care. The intention was to provide evidence on whether or not immediate and prolonged SSC between all mothers and babies at the time of birth was acceptable.

## Background

SSC is the placing of the newborn baby directly on its mother's skin immediately following birth. The baby is briskly dried and at once placed naked on its mother's skin, and then both the mother and the baby are wrapped in warm blankets. Strong evidence suggests that this simple, cost-free practice increases early feeding (Righard & Alade 1990; UNICEF 2013), particularly breastfeeding (Cattaneo & Buzzetti 2001; Kramer et al. 2001). SSC stabilizes the infant's metabolic system while at the same time increases maternal oxytocin level. In turn, this enhances maternal responses to the baby (Strathearn et al. 2009).

Yet there is evidence that some midwives are concerned about the contaminating secretions of birth: blood, amniotic fluid and mucus (Chesney 2009; Kirkham 2007). They feel a compelling need to ‘clean up’, and this is viewed as being the midwife's responsibility (Kirkham 2007). Sometimes, this desire for cleanliness supersedes the importance of supporting families, particularly helping mother and baby to develop a nurturing, intimate bond. This attachment between the mother and the infant starts in pregnancy and is built upon in the early post-natal period during periods of unrestricted SSC (Allen & Duncan Smith 2008, Centre for Excellence and Outcomes in Children and Young People's Services 2010, Strathearn et al. 2009). Both Sheridan (1999) and Price (2006) recognized that some midwives would resist change in practices that were supportive of SSC and would base their resistance on an argument that SSC was abhorrent to non-Westernized women and on the expectation of increased workload for the midwife. There is little research evidence, however, of the responses of women from different cultural backgrounds.

Traditionally, particularly across cultural groups organized around religion, blood associated with menstruation and birth is noted to be polluting, contaminating and unclean. This is the case for Islamic families, orthodox Jewish communities, Sikhs and Hindus (Shildrick 1997). However, in clinical practice decisions made are based on cultural stereotypes rather than individualized beliefs and expectations. It may be assumed without direct enquiry that when blood, amniotic fluid or vernix caseosa is present during SSC then intimate contact will be repugnant.

Cultural stereotyping remains widespread. McFadden et al. (2012) studied the impact of cultural context on the experiences of breastfeeding support for women of Bangladeshi origin, finding that the women's varied self-perception of their needs for breastfeeding support was at odds with the common assumption by practitioners of homogeneity and cultural barriers. Similarly, Sheau-Huey et al. (2008) carried out a prospective exploratory study in the USA with 48 healthy and culturally diverse mothers of full-term infants. Breastfeeding rates were observed to increase when SSC was used as an intervention for breastfeeding problems regardless of cultural grouping.

Byaruhanga et al. (2008) explored perceptions (though not experiences) of SSC with 30 post-birth mothers from a peri-urban area of Uganda during immediate SSC and infant care. Engagement in SSC was found to be dependent on social, cultural and economic factors rather than being influenced by the healthcare system. However, if women were informed of the benefits of SSC during pregnancy, they became ‘sensitized’ and more likely to participate in SSC, despite strong cultural beliefs that blood, vernix caseosa and amniotic fluids were dirty and contaminating. This suggests that engagement is at least partly influenced by healthcare interventions and that cultural barriers to birth fluids can be overcome.

The impact of separating a mother and infant is significantly demonstrated in the literature: inability to attune to the baby and to show love and affection (Bergman 2008; Gerhart 2004); reduced periods of breastfeeding (Cattaneo & Buzzetti 2001; Kramer et al. 2001); and poor regulatory responses in the infant (Chrisstenson et al. 1992; Moore et al. 2012; Saloojee 2008).

The need to promote SSC for all babies is vitally important, then, regardless of feeding mode and cultural background. Moreover, it is important for health professionals to lay aside assumptions of cultural belief and expected response to birth fluids in order to allow an informed free choice for mothers of all cultures.

## Study design

An interpretive, Heideggerian, phenomenological approach was adopted, guided specifically by the work of Benner (1994). Interpretive phenomenology is concerned with the interpretation of the structures of people's experiences and how things are understood by people who live through an experience. The study was idiographic, seeking to offer an insight into how a given person, in a given context, makes sense of a given phenomenon.

### Ethical approval

Approval for the study was secured from an NHS research ethics committee and from the University of Salford research ethics committee. Care was taken in composing information sheets in formats that could be understood by women from the three groups under study and in a way that would not cause offence or distress.

### Sample

Twenty women who participated in the study had experienced 1 h or longer of uninterrupted SSC immediately following birth. They were recruited during the last 3 months of their pregnancy from a hospital maternity unit in England and were drawn from three cultural groups: Bangladeshi, English and Pakistani.

### Data collection

Data collection by a variety of methods was completed in 2010. Digital recorders enabled participants to record a diary, video recordings shared visual data, and one participant used photography to capture and illustrate her experience. A great depth of understanding of phenomena can be gained using these methods. These activities empowered the women to reflect back to the time and situation and to record the associated memories, smells, sounds and feelings. Through this, they provided a rich perspective of that particular point in time. Video recorders were used by the partner or fixed on tripod stands to record the period of immediate SSC of four participants. This generated a large amount of visual data.

Interviews were conducted with the participants using the recorded data as a focus and prompt for discussion. Each interview lasted between 1 and 2 h and the whole of the data collection took 2 years to complete. Interviews were conducted at home or in the hospital, the organization's ethnic health team providing synchronous translation when required. The process of interviewing was iterative rather than linear so that learning from one encounter was carried into the next. The women spoke freely and passionately about their experiences. Observational data during review of the video footage and interviews were included in contextual field notes.

**Fig 1:**
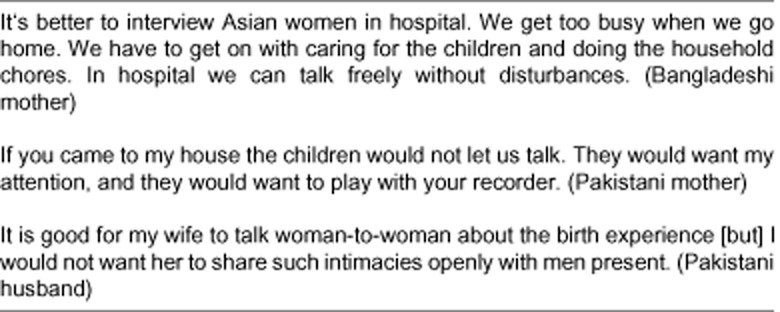
Asian women's preference to be interviewed in hospital.

Shelton & Rianon (2004) identified the difficulties in engaging South Asian women in research, suggesting that women from these communities were oppressed and often unable to share their voices. Although all participants were offered the same options, all of the Bangladeshi and Pakistani participants identified difficulties in undertaking video recording and opted instead to complete audio-diaries. They also preferred to be interviewed in hospital on the first or second post-partum day rather than at home (see Fig. 1).

### Data analysis

**Fig 2:**
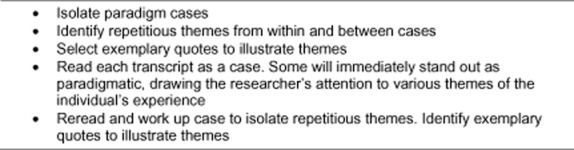
Steps for hermeneutic analysis (Benner 1994).

Analysis was cyclical, reflective and, at times, a messy process. When contextualized features of a lived experience are generated from a blend of meanings and understandings articulated by the researcher and participants, such as in this study, interpretive (Heideggerian) phenomenology provides the right framework (Parse 1999). Analysis began with reading and rereading participants' narratives to acquire a feeling for participants' ideas in order to understand and apply meaning to them. Benner (1994) proposes three interrelated processes in hermeneutic study, thematic analysis, analysis of exemplars, and the search for a paradigm, and these were pursued in this study (see Fig. 2).

The behavioural units in the video recordings included gaze, head turns, speed of approach to the breast, facial expressions, small shifts in arousal and mother–infant vocalization. Concept maps (Long & Johnson 2001) enabled the production of a visual map of the findings from the data. This enabled data to be displayed in a way that empowered a deeper understanding and facilitated further development of themes. The maps revealed what Stern (1977) termed the dance between the mother and the baby.

## Findings and discussion

In this study, mothers and babies clearly interacted with each other; the baby elicited responses from the mother, and the mother explored, touched, smelled and nurtured her infant intuitively. Yet, babies are not mere passive actors either. They are active communicators (as demonstrated in the video footage). They use systems of eye contact, cries and facial gestures to direct responses from their caretakers. This relationship, which develops during pregnancy and extends after birth, shapes and changes the brains of mother and infant.

### Contextualizing body fluids at birth

For some Asian women in the study, blood, mucus and amniotic fluid were found acceptable in the context of birth; yet, the same secretions were seen to be dirty, polluting and contaminating outside of this context. Aisha showed no revulsion to birth secretions coating her baby's skin. She was clear that this is what one would expect, it was quite normal, and in no way repulsive. ‘It doesn't cross your mind that you have your chest out and it is covered in mucus because it's your baby at the end of the day’ (Aisha. Bangladeshi. Hospital birth). Kashir also reflected on cultural aspects of birth, and suggested that blood and bodily fluids on a new baby would cause no cultural challenges to her. ‘He was all wet with blood on him, but he is part of me – just born. He was bound to have blood on him. It's not a problem, not an issue culturally either’ (Kashir. Pakistani. Hospital birth).

For Jayne, if the baby had been washed the natural aspect of her birth would have been lost. The time taken to separate and bath her baby would have impacted on how soon she could have held him. ‘I didn't care about the smell of the blood, the feel of him. In fact, I loved it. It made me realise what a beautiful and natural process birth is. I just wanted him, still covered in my blood. I didn't want him washed, I just wanted him’ (Jayne. English. Hospital birth).

Oxytocin, a hormone that has been strongly associated with both mothering and breastfeeding, is released in high levels immediately after the placenta is delivered. Physiologically, this hormone promotes immediate mothering actions, touch, gaze and vocalization. The need of a mother to hold her baby immediately may be influenced by the high levels of this hormone at the time of birth. Oxytocin also reduces the level of free-flowing catecholamines (stress hormones) in the baby (Henderson et al. 2011). It has been shown to evoke feelings of contentment, cause reductions in anxiety, and promote feelings of calmness and security. Elevated levels in the post-partum period have repeatedly been shown to enhance mother–infant attunement and bonding (Gordon et al. 2010).

Stern (1985) recognized that the infant's brain needs time to develop and mature. The baby regulates its inner world through aligning its state of mind with the caregiver. Largely through eye gaze, a conduit of empathic attunement is established. According to Stern, this conduit acts as an emotional umbilical cord that nurtures the child's emotional development. Therefore, the baby's relationship with his mother is crucial because it acts as a template for later emotional relationships. The baby arrives in the world with an ability to mimic facial expressions. This ability has been recorded as early as 10 min of age (Stern 1985). The mother's face and its animation are crucially important to the infant. The emotions of the infant direct what the mother does as much as the mother's emotions direct the infant. ‘Their loop operates in both directions, a primal emotional highway’ (Goleman 2006, pp. 163–164).

### Birth without skin-to-skin contact

Some women referred to their previous birth experiences without SSC. ‘I had thought about it, him being slimy and wet, horrible and messy.’ Lisa went on to explain that ‘In comparison to this birth with immediate SSC, my first birth now appears clinical and cold. He was all washed, clean; there was none of that nice smell about him that you associate with birth. It's that what makes you want to hold them, to get to know them’ (Lisa. English. G2P2. Home birth). Lisa did not experience the ‘messiness’ of birth that she had anticipated and she now ascribed positive associations to SSC. ‘I can't imagine delivering him and twenty minutes later being presented with a pristine baby. I would feel alienated by that, whereas this, a wet, bloodied baby feels like the most natural thing in the world.’

In a previous small study (Finigan & Davies 2004), Hannah had described her medicalized birth experience in a similar way: ‘I can only say that if SSC was not available, birth would be clinical. We would be like subjects of some hospital or medical routine. You push, baby is delivered, whisked away, checked over, wrapped and given back to you, and then you are whisked off to some postnatal ward.’ This description of birth without SSC conjures up a system that is similar to a conveyor belt or production line, with birth being processed clinically and impersonally: a sad reflection of failure of women-centred midwifery care.

### Multisensory facets of birth fluids: touch and smell

Kirkham (2007) accepted that ‘birth dirt’ exists, referring to the greasy vernix coating, the blood and mucus, and meconium and urine that the baby may excrete during the birth process. She also recognized birth smells: distinct yet not offensive that are unique to birth. Viv mentioned these secretions in her diary but showed no revulsion to them. ‘A bit messy really. She did her first poo on me; this will be the first of many’ (Viv. English. Home birth). No abhorrent behaviours were seen in the video footage. Val was shown minutes after birth, pulling the stretchy, tenacious membranes from her baby's head. She held them up and looked at them inquiringly. There was no expression of distaste upon her face. She passed the membranes to her husband who, in turn, explored them. Then, the membranes no longer holding any interest were placed in the bin. In the video recording, it was clear that the newborn baby was covered in speckles of blood and mucus, and thick, heavy vernix coated her skin. Regardless, mother, father and siblings gently touched and kissed the baby, paying no attention to the messiness.

The women's stories suggest that a mother's caregiving repertoire need not be taught. Indeed, Stern (1977, p. 11) argues that ‘it cannot be taught, but it can be disinhibited’. Delaying the start time for SSC and limiting its duration may be an inhibiting factor of natural motherhood which otherwise has lasting impact. ‘There was a unique smell to him, the texture of his skin. It was fantastic. That kind of unique smell will link me to that time forever’ (Val. English. Home birth).

### Differing causes of the response to birth fluids

Douglass (1996) concludes that ‘matter’ when seen out of place becomes unacceptable. This perspective was observed in the study. ‘Bodily fluids, secretions at birth, were natural for me, when they were on the baby, that is, but not when they were smeared on me. That was different’ (Kashir, Pakistani. Hospital birth). Kashir felt shame. She was ‘unclean’ when blood was smeared on her nightwear with her family present. The cultural belief that birth blood is contaminating and unclean now became apparent. Bharj (2007) noted that women are considered to remain unclean until after they are ritually cleansed. Menstrual blood, lochia (blood following birth) and breastmilk are all considered to be polluting in many cultures. Jeffery et al. (1988) noted the work of the Dai (a local uneducated woman in Pakistan who delivers babies), reporting the widespread belief that this was defiling because the perceived pollution associated with childbirth was considered to be far worse than other sources of pollution such as menstruation or defecation.

A second Pakistani mother reported that ‘If he had been wet from birth then I wouldn't have minded, wouldn't have been bothered, but blood … It's just the blood I don't like. Other than that you just love your baby’ (Preetia. Pakistani. Caesarean birth). In contrast to Kashir's account, this mother simply had a phobia of blood, which could affect any woman and was not culturally significant. Sakina also did not find birth secretions to be culturally or personally distasteful. She perceived no religious issues about her baby being unwashed or unclean. However, she did explain that the water on the baby made it difficult to hold: a purely practical problem. ‘Not really – it's your blood and gunge, but there is water on the baby, and you're scared it will slip from your hands.’ She reflected on a previous birth: ‘if I had been given a choice, I would have asked them to dry my baby. She was slippery and difficult to hold that was my only problem’ (Sakina. Bangladeshi. Hospital birth).

The women's words suggest that true cultural care must be individualized, without assumptions of universal response with a cultural group. What is welcomed by one mother may be distressing for another, and for many different reasons. These findings contrast sharply with previous suggestions about birth fluids being inevitably associated with cultural abhorrence (Billings 1995; Eyers 1992).

## Implications for nursing and health policy

The findings from this study offer messages for changes in professional practice, in local policy and in national strategy.

### Implications for professional practice

The implications for professional practice relate specifically to midwives in this study, but the messages can be seen to be of relevance to other professionals and other fields of practice, too, particularly with regard to being more open to the expressed preferences of service users. The findings demonstrate the potential for erroneous assumption on the part of health professionals, reminding that women from any community, religion or culture may have a phobia of blood or be frightened that a slippery baby may wriggle from their hands. Professional practice must incorporate caution in acting upon unverified assumptions based on cultural background.

For the majority of south Asian women in this study (though not all), otherwise unacceptable fluids associated with birth were accepted in the specific context of birth. This means that midwives need to consider women's feelings about SSC and birth fluids on an individual basis and be respectful of their wishes. Some women may welcome birth fluids on their babies' skin and view them as a natural part of their birth experience. Other women may prefer secretions to be wiped from their babies' skin, or they may want their babies bathed before SSC begins. Such choices must become routine in the practice of midwives, nurses and other health professionals.

### Implications for local policy

This study provides a compelling rationale for changes in labour ward practices to promote environments that enable mothers, babies and fathers to function as a nurturing unit, maximizing the opportunities afforded by hormonal changes at birth, and promoting choices unfettered by cultural stereotyping. For change in practice to occur, health-providing organizations must adopt local policies to ensure that choices are made clear and are available to patients, and they must provide training and leadership in responding in a supportive mode when situations are culturally challenging for service users.

### National policy

Practices that support breastfeeding are embedded into national policy strategies in the UK in order to improve population health and to tackle health inequalities (NICE 2012). UNICEF (2012) reported that moderate increases in breastfeeding would translate into cost savings for the NHS of at least £40 million per year, with tens of thousands fewer infant admissions to hospital and fewer general practitioner visits for infant illnesses if babies were breastfed. Effective implementation of UNICEF's Baby Friendly standards clearly increases the initiation and continuation of breastfeeding (Cattaneo & Buzzetti 2001).

Step 4 of the standards highlights that immediate and continued SSC between the mother and the baby promotes early and instinctive feeding and appears to lead to more confident parenting. However, some maternity units are challenged by step 4. Dykes (2006, p. 72) alludes to the pressures within the labour ward and post-natal setting as ‘a production line’: a place where midwives are expected to provide for other's needs in a culture that ‘races against the clock’. There is little time for midwives to promote SSC in the labour wards as a means for breastfeeding or for the mother and baby relationship to begin.

The midwives involved in caring for women in this study were innovative and found ways around this. For example, if the labour ward rooms were busy, they transferred the mother and the baby in SSC to the post-natal wards and continued to keep them together. The midwives' tenacity in supporting mothers' choices demonstrates that the midwives understood the meanings and values that each woman was bringing to her own particular early experience of motherhood.

This study provides evidence that SSC is not abhorrent to women from diverse population groups which has been a key issue for implementation of the standards in some UK maternity units where there is a large ethnic population. This study and others, for example, Velandia et al. (2010), highlighted the need for longer periods of uninterrupted SSC which should continue until the baby has at least taken its first feed and be discontinued only at the mother's request.

## Limitations

The study was conducted in a single setting, eliciting individual experiences of participants who may not be representative of others in their cultural groups nationally. However, their collective narrative and recordings may find resonance and have implications for midwifery practice in other parts of the world. The need for third-party translation during interviews with Asian women may have added a layer of interpretation to data analysis. The ability of third parties to exclude some groups from participation was underestimated. This occurred through imposition of cultural expectations in the recruitment process by peer group members, a process that was amended once the issue came to light.

## Conclusions

Stereotypical assumptions about cultural background rather than recognition of women's individual beliefs and needs often characterize professional responses. In this study, once this stereotyping was neutralized, women from all three cultures were seen to enjoy intimate SSC with their newborn babies. This was the case whether they had chosen breastfeeding or bottle-feeding for their babies. This study demonstrates that midwives should take a non-judgemental approach to understanding the choices that women make, particularly when these may not fit into the institutional guidance and protocols.

## Author contributions

VF: Study conception, data collection. VF and TL: Study design, data analysis, drafting the manuscript. TL: Critical revisions for intellectual content, supervision.
